# Expression of Active Fluorophore Proteins in the Milk of Transgenic Pigs Bypassing the Secretory Pathway

**DOI:** 10.1038/srep24464

**Published:** 2016-04-18

**Authors:** Ayan Mukherjee, Wiebke Garrels, Thirumala R. Talluri, Daniela Tiedemann, Zsuzsanna Bősze, Zoltán Ivics, Wilfried A. Kues

**Affiliations:** 1 Friedrich-Loeffler-Institut, Institut für Nutztiergenetik, Mariensee, Germany; 2 Medical School Hannover, Institute of Laboratory Animal Sciences, Hannover, Germany; 3 NARIC- Agricultural Biotechnology Institute, Gödöllö, Hungary; 4 Paul-Ehrlich-Institute, Langen, Germany

## Abstract

We describe the expression of recombinant fluorescent proteins in the milk of two lines of transgenic pigs generated by *Sleeping Beauty* transposon-mediated genetic engineering. The *Sleeping Beauty* transposon consisted of an ubiquitously active *CAGGS* promoter driving a fluorophore cDNA, encoding either Venus or mCherry. Importantly, the fluorophore cDNAs did not encode for a signal peptide for the secretory pathway, and in previous studies of the transgenic animals a cytoplasmic localization of the fluorophore proteins was found. Unexpectedly, milk samples from lactating sows contained high levels of bioactive Venus or mCherry fluorophores. A detailed analysis suggested that exfoliated cells of the mammary epithelium carried the recombinant proteins passively into the milk. This is the first description of reporter fluorophore expression in the milk of livestock, and the findings may contribute to the development of an alternative concept for the production of bioactive recombinant proteins in the udder.

For large scale production of recombinant proteins, the mammary gland of mice and livestock has been exploited extensively as bioreactor^1^,^2^,^3^. In principle, recombinant proteins can be obtained by milking of transgenic animals^4^,^5^,^6^, and high yields in the scale of grams per liter have been obtained for human lactoferrin^7^,^8^,^9^, alpha-lactalbumin^10^, acid alpha glucosidase^11^, albumin^12^ and lysozyme^13^.

The first transgenic livestock were developed in 1985^14^. Since then several attempts have been carried out to produce recombinant proteins in livestock. This includes the generation of transgenic pigs for production of bovine alpha lactalbumin^15^, human factor VIII^16^, recombinant human factor IX^17^, or human lysozyme^18^ in the udder. The generation of transgenic cattle allowed for the increased production of β- and κ-caseins^19^, human lactoferrin^7^, lysostaphin^20^, or of trans-chromosomic cattle for the production of human antibodies in serum^21^. Transgenic goats were established for udder-specific expression of human lysozyme^22^,^23^, human anti-thrombin III^24^ or recombinant butyrylcholinesterase^25^. Transgenic sheep expressing human factor IX^26^, and transgenic rabbits expressing insulin-like factor I and human acid alpha-glucosidase^11^,^27^ have been established.

Currently, the first drugs from the milk of transgenic goats and rabbits, which are approved for human treatment by the European Medicines Agency (EMA) and the American Food and Drug Agency (FDA) are recombinant anti-thrombin and C1-esterase^28^,^29^,^30^.

However, in a number of attempts only minute amounts of recombinant proteins could be detected in the milk of transgenic animals^31^,^32^,^33^. Typically, mammary gland specific promoter and regulatory elements, such as casein, lactoglobulin and lactoalbumin promoter elements were used to target the expression of a recombinant protein to the mammary epithelium during the lactation period. Secretion of the recombinant protein into the milk requires an amino-terminal signal peptide, which directs the nascent polypeptide into the endoplasmic reticulum. Via the Golgi-apparatus, the matured proteins are transported into secretory vesicles, which fuse with the cell membrane and release their cargo into the lumen of the mammary gland.

Here, we describe a radically different approach to achieve high levels of recombinant proteins in the milk of transgenic pigs. Previously, we employed the *Sleeping Beauty* (SB) transposon system to generate germline-transgenic pig lines with reporter transposons encoding *Venus* and *mCherry* fluorophore cDNAs, respectively^34^. Both reporters were driven by the ubiquitously active chimeric cytomegalovirus (CMV) enhancer and chicken β-actin promoter (*CAGGS*). The Venus and mCherry transposons were designed for cytoplasmic expression of the recombinant proteins. Indeed, previous analyses of organs, tissues and cultured cells from these animals confirmed this prediction^34^,^35^,^36^,^37^. Here, we analyzed the milk of lactating transposon sows, albeit the design of the transgenic construct did not include a signal peptide for the secretory pathway, which is thought to be critical for the transport of recombinant proteins into the milk. We show that both reporter lines secrete high levels of recombinant Venus and mCherry proteins into the milk. Importantly, the present findings could emerge as an alternative approach to produce bioactive proteins in the udder.

## Results

### Expression of Venus fluorophore in sow milk

**Figure 1:**
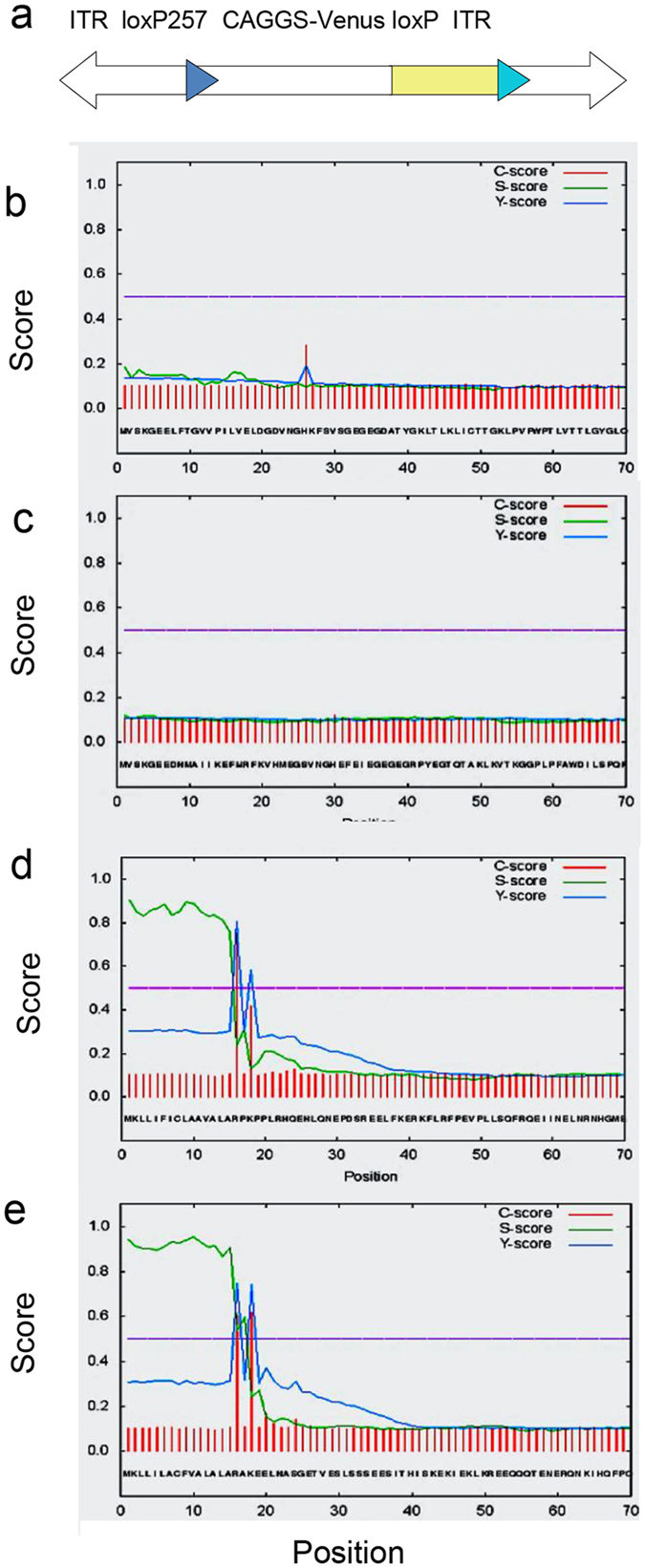
*In silico* prediction of signal peptides. (**a**) Design of transposons. The *CAGGS* promoter driven cDNA is flanked by heterospecific loxP sites and the SB inverted terminal repeats (ITR). Here the *Venus* transposon is depicted, the *mCherry* transposon has an identical design. (**b,c**) Signal peptide analysis of the Venus and mCherry coding sequences. Note, that the algorithm does not predict a signal peptide for Venus or for mCherry. (**d,e**) Signal peptide analysis of porcine alpha s1 (GenBank: EU025875.1) and beta caseins (GenBank: EU213063.1). Note, the distinct prediction of signal peptides in the milk proteins. C-score, raw cleavage site score; S-score, signal peptide score; Y-score, combined cleavage site score. For further details see SignalP4.1 website (www. http://www.cbs.dtu.dk/services/SignalP/output.php).

**Figure 2:**
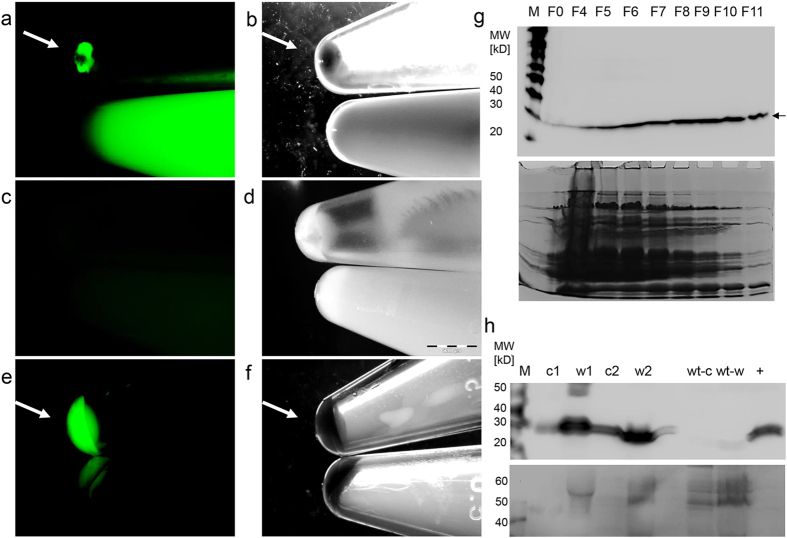
High level expression of Venus in transgenic milk. (**a**) Sedimented milk cells (top) and skimmed milk (bottom) from a *Venus* transgenic sow shown under specific excitation of Venus (50 ms exposure). Arrow points to pellet of milk cells at the bottom of a 1.5 ml centrifugation tube (isolated from 1 ml of milk). (**b**) Corresponding brightfield illumination. (**c**) Wildtype milk cells (top) and skimmed milk fraction shown under specific Venus illumination. (**d**) Same samples as in (c) shown under brightfield illumination. (**e**) Milk cells (top, arrow) and fat fraction (bottom) of milk from a *Venus* transgenic sow shown under specific excitation of Venus. Note that the fat fraction displays a reduced fluorescence relative to the cell pellet. Arrow points to cell pellet. (**f**) Same samples as in (e) shown under brightfield illumination. (**g**) Top: Venus immunoblot of fractions elution from a Sephadex G50 column loaded with Venus containing skimmed milk. M, size marker (Magic Mark); F0 before loading on column; F4-F11, collected fractions of flow-through. Arrow indicates Venus protein, migrating at an apparent molecular weight of 29 kD. Bottom: corresponding Coomassie-stained gel. (**h**) Immunoblots of Venus (top) and tubulin (bottom) from milk cells (c1, c2) and skimmed milk (w1, w2) fractions of two different samples of Venus-containing milk, and from milk of a wildtype sow (wt-c, wt-w); +, positive control of purified Venus; M, size marker (Magic Mark).

A bioinformatic analysis predicted no secretion signal sequence in the Venus or mCherry sequences (Fig. 1), whereas a clear signal peptide prediction was obtained for known porcine milk proteins, like alpha s1 casein and beta casein. Nevertheless, milk samples collected from lactating transposon-transgenic sows contained high levels of the respective recombinant reporter proteins, which could be readily identified by fluorescence microscopy (Fig. 2). In total, milk samples were collected from two *Venus* transposon sows, three *mCherry* transposon sows and five control (non-transgenic) sows.

In Fig. 2 an exemplary milk sample from a *Venus* transposon sow is shown under specific excitation in a stereozoom fluorescence microscope. Both the milk cells (concentrated from 1 ml milk) and the skimmed milk fraction contain high levels of Venus fluorophore protein, whereas a milk preparation from a control animal did not show any specific fluorescence under identical conditions (Fig. 2).

A direct fluorescence comparison of skimmed milk and fat fractions suggested that the skimmed milk contained much higher Venus levels, excluding the possibility that the Venus protein was somehow secreted through the fat micelle pathway. The absence of a consensus sequence for a signal peptide suggested that the Venus protein was not secreted via the endoplasmic reticulum. Another possibility is that exfoliated cells from the mammary epithelium may have carried Venus protein trapped in their cytoplasm into the milk. To assess this scenario, immunoblots of skimmed milk and cell fractions were done with antibodies against Venus and against ß-tubulin, a typical cytoskeletal protein. The cytoskeletal ß-tubulin could be detected in the skimmed milk fractions (Fig. 2), suggesting that a certain fraction of milk cells become membrane-damaged in the udder lumen and release their content into the milk. However, the tubulin signals in the milk samples showed a high variability, which seem to reflect degradation processes and individual differences between the milk donors.

**Figure 3:**
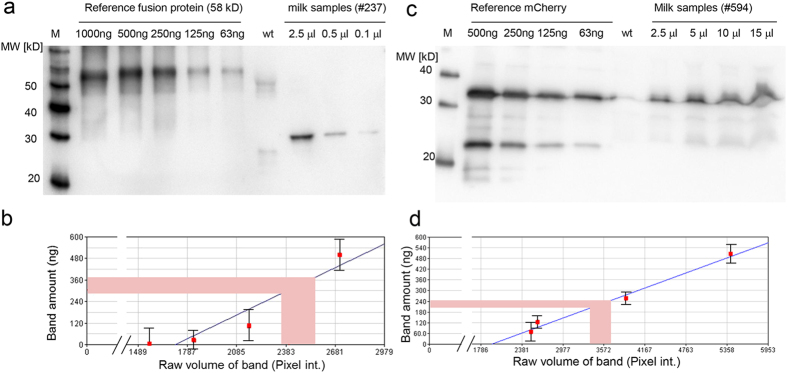
Determination of fluorophore concentrations in milk of transgenic sows. (**a**) Different concentrations of a recombinant GFP-fusion protein (58kD) were loaded alongside of milk samples from a wildtype (wt) and a Venus transposon sow (#535) on a 12% SDS-PAGE, blotted to a PVDF membrane, and probed with an anti-EGFP antibody. (**b**) Densitometric analysis of blot suggesting a Venus concentration of 0,27–0.38 g/l in milk from transgenic sows. (**c**) Different concentrations of a recombinant mCherry protein were loaded alongside of milk samples from a wildtype (wt) and a mCherry transposon sow (#594) on a 12% SDS-PAGE, blotted to a PVDF membrane, probed with an anti-mCherry antibody, and densitometrically evaluated. (**d**) Densitometric analysis suggesting a mCherry concentration of 0.20–0.25 g/l in milk from transgenic sows.

The skimmed milk fraction from Venus transposon sows could be used to enrich the Venus protein by size chromatography (Fig. 2, Supplementary Fig. S1). The content of recombinant Venus protein was determined to be between 0.27–0.38 g/l of milk (Fig. 3).

### High level expression of mCherry fluorophore in the milk

**Figure 4:**
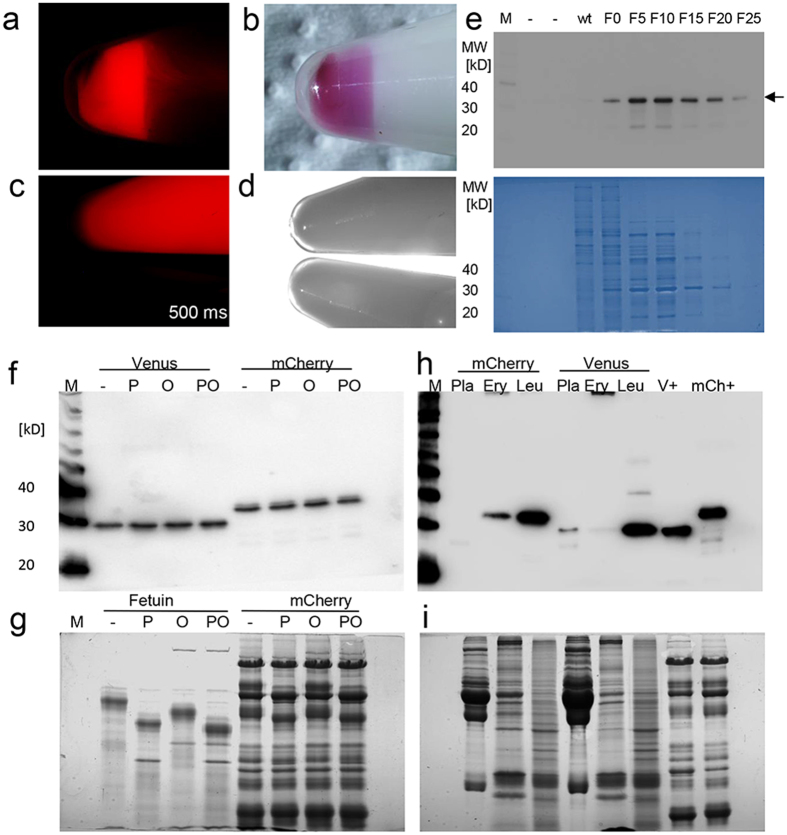
Large scale expression of mCherry in sow milk. (**a**) Milk sample from a *mCherry* transgenic sow shown under specific excitation of mCherry (15 ms exposure). The milk cells were sedimented by centrifugation at the bottom of a 15 ml tube (concentrated out of 15 ml milk). Note the apparently higher expression of mCherry in the cells compared the skimmed milk. (**b**) Same sample shown under brightlight illumination. The high mCherry content colored the milk cells purple/reddish. (**c**) Skimmed milk fractions of milk from a *mCherry* (top) and a wildtype sow (bottom). At extended exposure times (here 500 ms), the mCherry could also be detected in the skimmed milk fraction. (**d**) Corresponding brightfield view of (**c**). (**e**) Top: Immunoblot of mCherry in protein fractions of milk cell extracts separated via an anion exchange column. M, size marker; -, empty slot; wt, wildtype milk cells; mCherry milk cells; F0–F 25, fractions 0 to 25. Arrow point to mCherry protein, migrating at an apparent molecular weight of 33 kD. Bottom: Corresponding Coomassie stained gel. (**f**) Absence of glycan-residues in Venus and mCherry proteins from sow milk. Immunodetection of Venus and mCherry. M, size marker; -, untreated milk proteins; P, PNGase treated; O, O-glycosidase/neuramidase treated; PO, PNGase and O-glycosidase/neuramidase treated. (**g**) Deglycosylation of fetuin control, and total milk proteins, same labels as in (**f**), Coomassie stained gel. (**h**) Absence of Venus and mCherry in blood plasma from transgenic sows, as determined by immunodetection. M, size marker; Pla, plasma; Ery, erythrocytes; Leu, leucocytes; V+, Venus positive milk sample; mCh+, mCherry positive milk sample. (**i**) Corresponding Coomassie stained gel of (**h**).

Subsequently, the milk from three *mCherry* transposon sows was analyzed. In the milk cell fractions, the mCherry fluorophore was readily detectable (Fig. 4), actually the high mCherry content colored the milk cells (here concentrated from 15 ml milk) reddish under white light illumination. The skimmed milk fraction apparently contained lower mCherry concentrations than the pellet of the milk cells (Fig. 4), supporting the notion that indeed the somatic milk cells carried the mCherry protein in a piggyback manner into the milk. The mCherry from extracted milk cells could be enriched via column purification and expression could be confirmed by immunoblotting with an anti-mCherry antibody (Fig. 4). The content of recombinant mCherry protein in milk from the transgenic sows was determined to be 0.20–0.25 g/l (Fig. 3c,d).

**Figure 5:**
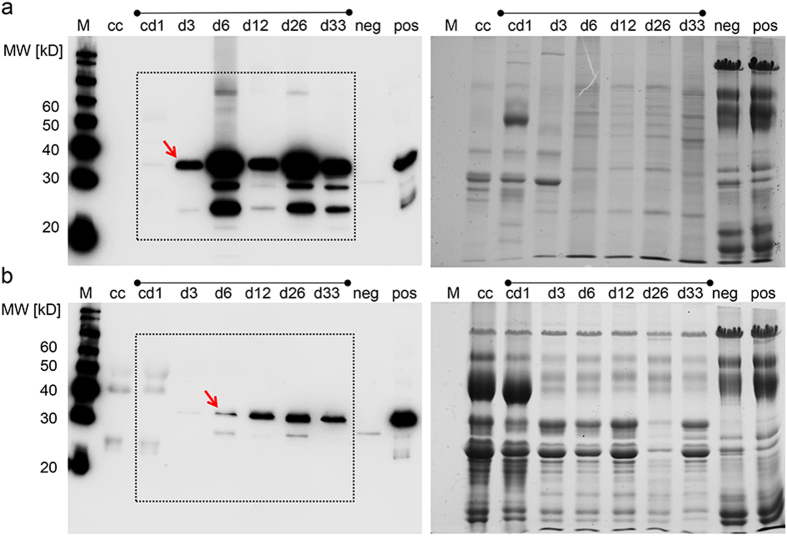
Time course of reporter expression during lactation. (**a**) Expression of mCherry in somatic cells and (**b**) skimmed milk, collected from colostrum (cd1), and at days 3, 6, 12, 26 and 33 (d3-d33) of lactation; M, size marker; cc, control colostrum from wildtype sow; neg., negative milk control; pos, positive milk control (from mCherry sow). Left immunoblot with anti-mCherry antibody, and right corresponding Coomasssie stained gel (loading control). Note that the colostrum of the transgenic sow (cd1) did not show detectable mCherry expression. In the somatic cells the mCherry signal comes up at d3 (arrow), and in the skimmed milk at day 6 (arrow). The signal intensity is normalized to the marker bands and the positive control, suggesting that the majority of mCherry is deposited in the somatic cells, and released to the skimmed milk by membrane-damaged cells.

A time-course experiment to follow the expression of the reporter proteins from day 1 of lactation (colostrum) to weaning is shown in Fig. 5. Interestingly, in colostrum no expression of the reporter (mCherry) could be detected. The mCherry expression started in the somatic cells from day 3 and was then detectable at high levels until weaning. In the skimmed milk, expression of mCherry was first detectable at day 6 and was then continuously present until weaning, but in lower levels than in the somatic cells (Fig. 5). The total amounts of exfoliated cells were similar in the milk from transgenic and control wildtype sows (Supplementary Fig. S2).

### Analysis of potential glycosylations and secretion into blood plasma of Venus and mCherry

To substantiate the proposed transport of Venus and mCherry protein via exfoliated cells into the milk, it was analysed whether the recombinant reporters were post translationally modified by N- and O-glycosylations, which are typically occurring during processing in the secretory pathway. The treatment of skimmed milk fractions of *Venus* and *mCherry* sows with deglycosylating PNGase F and O-glycosidase did not show any evidence for glycosylations (Fig. 4f,g), despite the bioinformatic prediction of potential O-glycosylation sites on Venus (aa85) and on mCherry (aa136), and several potential N-glycosylation sites.

To test whether the transposon transgenic pigs bear the reporter proteins in other body fluids than milk, blood was sampled, separated into plasma, erythrocyte and leucocyte fractions, and used for immuno-detection of Venus and mCherry. Typically, the recombinant protein could not be detected in the plasma fractions (Fig. 4h, Supplementary Fig. S3). Only in one sample a minimal amount of Venus could be detected, which more likely represent a case of cell lysis during blood sampling (Fig. 4h). In summary, these data support the cytoplasmic localization of the reporter proteins, the higher clearance rate of apoptotic or damaged cells from the blood circulation seems to prevent an accumulation of the reporters in the plasma.

## Discussion

Here, we describe the expression of recombinant reporter proteins in the milk and milk cells of two lines of transposon transgenic pigs. The transposon constructs were designed for ubiquitous expression and cytoplasmic localization of the encoded Venus and mCherry proteins, and previous studies proved this intracellular localization^34^,^37^. However, high levels of Venus and mCherry proteins were readily detected in sow milk samples from the two lines, respectively. For the Venus fluorophore an expression level of 0.27–0.38 g/l, and for mCherry an expression level of 0.20–0.25 g/l were determined. Considering that porcine milk contains 6–8 g of protein per liter^38^, this equals to 2.5–6.3% of the total protein content.

Our data suggested that the recombinant Venus and mCherry are not transported via the secretory pathway, but that exfoliated cells of the udder epithelium carried Venus or mCherry into the milk. During lactation, the udder epithelium represents one of the most highly proliferative tissues in mammals^39^, and undergoes substantial remodelling^40^. The somatic cell count of porcine milk from healthy sows has been determined between 10^8^ to 10^10^ cells per liter^41^,^42^, which is a much higher than for example in cow milk (0.009 × 10^9^ cells per liter)^43^.

The milk cells represent apoptotic cells, but also vital lactocytes (milk secretory cells), epithelial and immune cells^44^. The colostrum typically contains a relatively high number of immune cells, which declines in mature milk. In healthy sows after the first week of lactation, the vast majority (>95%) of milk cells consist of lactocytes and mammary myoepithelial cells^42^. The analysis of milks samples during lactation reveals important aspects with regard to the expressing cells. First, in the colostrum predominantly reporter-negative cells are present, and second during transition to normal milk, reporter-positive cells appear, followed by a delayed appearance of the reporter protein in skimmed milk. Colostrum from sows contains primarily leukocytes and within the first week of lactation the ratio of lactocytes and epithelial cells increases drastically^43^. The difference of reporter expression in the colostrum cells to leucocytes isolated from blood suggests that a specific sub-fraction of leucocytes contributes to colostrum formation. However, this warrants a more detailed analysis beyond the scope of this manuscript. Here we show that this phenomenon allows a simple enrichment of cytoplasmically-localized recombinant proteins by sedimentation of milk cells. We speculate that this principle may be useful for udder expression of other recombinant proteins, too.

A cytoplasmic localization of recombinant protein in mammary epithelial cells does not allow for N- and O-glycosylations, which take place inside the endoplasmic reticulum and the Golgi apparatus. However, several previous studies showed that mammary gland expression of recombinant proteins may result in faulty glycosylation patterns, for example variation in glycosylation has been observed in recombinant human lactoferrin produced in bovine^8^ and murine^45^ milk. Lower levels of sialylation and fucosylation have been found in recombinant human C1 inhibitor produced in transgenic rabbit milk^46^. Altered N-glycan patterns have been observed in recombinant human C1 inhibitor^46^, and recombinant human factor IX^17^. Importantly, an alternative endo-mannosidase pathway is present in the mammary gland of different livestock species, which is responsible for different manno-oligosaccharide patterns of the recombinant proteins^47^. This altered oligosaccharide and N-glycan patterns are thought to be immunogenic to humans^48^,^49^,^50^. Incomplete γ-carboxylation and inadequate endoproteolylic processing have been observed for different recombinant clotting factors and protein C^51^ isolated from the milk of transgenic animals.

Thus the described transport of recombinant proteins “trapped in the cytoplasm” of milk cells may be of interest for proteins, which do not require glycosylation for biological activity^52^, and for proteins, which can be glycosylated *in vitro*^53^. One particular advantage of cytoplasmic expression in the mammary epithelia is that a recombinant protein is protected from degradation processes occurring in the udder lumen, or from aggregation with fat micelles, which may complicate the purification^54^,^55^,^56^.

Commonly used promoters for expression in milk are alpha s1 casein^57^, beta casein^58^,^59^, whey acidic protein (WAP)^16^, and beta-lactoglobulin promoter^60^,^61^. Despite the employment of these milk-specific promoters, several recombinant proteins could only be produced in minute amounts^23^,^31^,^32^,^33^,^62^. Interestingly, also hybrid promoters consisting of fusion constructs of the *CMV* enhancer and milk protein promoters, such as ovine beta casein, or bovine alpha s1 casein have been found to result in increased expression of human lactoferrin in the milk of transgenic mice^63^. In AAV (adeno-associated virus)-transduced mammary epithelia of mice and rabbits the ubiquitous CAG promoter resulted in high expression of recombinant myelin basic protein^64^.

Generally, production platforms based on microbes and mammalian cells set the standards for the production of recombinant proteins, however the here described large scale production of non-glycosylated proteins in the udder may fill a niche that conventional system may not address, for example it is anticipated that the production of recombinant proteins in the milk may be more cost efficient due to low running costs^61^.

Here, we showed that ubiquitously expressed Venus and mCherry fluorophore proteins could be harvested at large scale in the milk of transposon transgenic sows. Genomic integration of transgenes by the SB transposase into transcriptionally permissive loci allows the reduction of the number of experimental animals and contributes to robust transgene expression levels^65^. Importantly, the SB system has been established for successful transposition in a range of mammals suitable for milking^35^,^66^,^67^.

**Figure 6:**
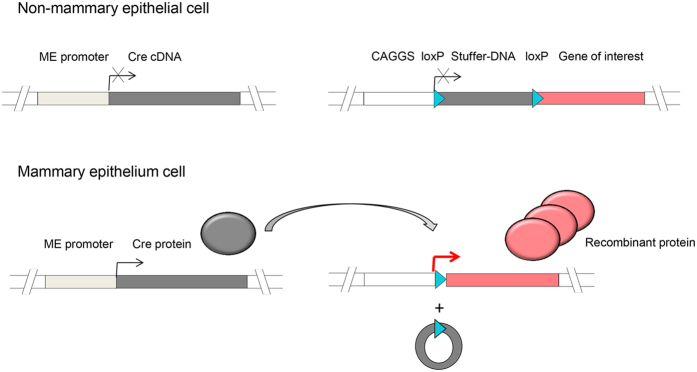
Concept for high level mammary epithelium-specific expression of recombinant proteins driven by a ubiquitously active promoter. The recombinant protein is separated by a floxed stuffer DNA sequence from the *CAGGS* promoter. The stuffer DNA contains stop codon sequences and thereby suppresses expression. On a second element a mammary epithelium (ME)-specific promoter drives a Cre cDNA. Only in mammary epithelium cells the *Cre* cDNA is transcribed, and activates the recombinant protein construct by Cre-mediated deletion of the stuffer. As a result, high level expression is restricted to mammary epithelial cells.

For recombinant proteins, such as growth factors, where ectopic expression outside the udder is undesired, the here describe system may be refined by combining it with the Cre recombinase system. A construct with a milk promoter driving the Cre-recombinase and a second construct with a *CAGGS* promoter, separated by a loxP flanked stop cassette from the gene of interest, would restrict the expression to the mammary gland, but still benefit from the strong transcriptional activity of the *CAGGS* promoter (Fig. 6). The proposed constructs may be combined on one cassette, or used in a binary approach. Recently, we established a simple and efficient multiplexing approach for transgenesis in cattle^68^, showing that advanced genome engineering techniques are nowadays sufficiently developed to perform this kind of gene transfer in livestock.

In sum, we established the cytoplasmic production of recombinant proteins driven by a strong ubiquitous promoter as an alternative approach for high level expression in milk of transgenic sows. This principle could emerge as an alternative and robust concept for large scale production of recombinant therapeutic proteins in the mammalian udder.

## Material and Methods

### Ethics statement

Animals were maintained and handled according to German and international laws regulating animal welfare and genetically modified organisms (GMO), and the experiments were approved by an external animal welfare committee at the Niedersächsisches Landesamt für Verbraucherschutz und Lebensmittelsicherheit in Oldenburg, Germany (AZ 33.9-42502-04-09/1718).

### Development of transgenic animals

*Venus* and *mCherry* transposon pig lines have been developed previously^34^. Briefly, the Venus positive porcine line has been generated by co-injection of pT2/RMCE-Venus transposon and pCMV-SB100X transposase plasmids into the cytoplasm of porcine zygotes. The mCherry positive porcine line has been developed by a targeted recombination-mediated cassette exchange (RMCE) of Venus against mCherry cDNAs^34^,^65^.

### Fractionating milk samples and Western blotting

Milk samples were collected from two *Venus* transposon and three *mCherry* transposon sows. As control, milk was collected from five non-transgenic sows. The piglets were removed from their mother 30 minutes before milking. The sows were then treated with novacen (Pharma Partner, 500 mg/ml, 10 ml, i.m.) and ten minutes later with oxytocin (Pharma Partner, 10 U/ml, 2 ml, i.m.), the milk was collected by manual stimulation of the teats. Immediately after collection, milk samples were incubated in ice and diluted 1:1 with phosphate buffered saline (PBS). Upon centrifugation at 500 g at 4 °C for 10 minutes, the milk was separated in milk cells (sediment), skimmed milk and fat fractions. Total protein was isolated from milk cells and skimmed milk, and subsequently SDS-PAGE was done as described before^69^. Polyclonal rabbit anti-GFP (Thermo (Life Science), CAB4211, 1:2000) and anti-mCherry (Biomol, 600-401-P16, 1: 5000) primary antibodies were used to detect Venus and mCherry proteins with a horseradish peroxidase-coupled anti-rabbit secondary antibody (1:10 000) (Sigma-Aldrich) and an enhanced chemiluminescence reagent (ECL plus; GE Healthcare). For chemiluminescence detection the Fusion FX Station (Vilber-Lourmat) was used. Alternatively, a monoclonal anti-GFP antibody (Developmental Studies Hybridoma Bank (DSHB), 12AG, 1: 500) was used in combination with an anti-mouse IgG (Sigma-Aldrich, 1:20000) conjugated to peroxidase. For detection of tubulin, the blot was stripped and re-probed with an anti-tubulin antibody (DSHB, E7, 1:500), followed by a secondary anti-mouse IgG-peroxidase incubation.

To determine the total amount of the reporter proteins in the transgenic milk, dilution series of a recombinant GFP (Santa Cruz, sc-4304 WB) and a recombinant mCherry N-His tag (BioCat) were used. As molecular size marker, Magic Mark XP (Novex, LC5603) containing proteins of 20, 30, 40, 50, 60, 80, 100, 120 and 220 kD weight with affinity to most secondary antibodies, was used. The chemiluminescence images were quantified by GelAnalyzer software (www.gelanalyzer.com). In brief, the quantity values for reference band of known concentrations were measured, and used to determine the concentrations of the reporter proteins in milk samples. For detection of total protein, the SDS page gels were stained with a Coomassie containing Page Blue Protein Staining (Thermo, 24620) according the manufacturer’s instructions.

### Fluorescence microscopy and image handling

For fluorescence microscopy, a Zeiss Axiovert 35 M stereozoom microscope equipped with epifluorescence filter modules for Hoechst 33342, EGFP/Venus (excitation 460–490 nm, dichroic mirror DM500, band pass filter 515–550 nm) and rhodamine (excitation 520–550 nm, dichroic mirror DM560, long pass filter 580 nm), and a high resolution digital camera (Olympus DP72), controlled by the CellF imaging software (Olympus), were used. Typically exposure times between 15 ms and 100 ms were used for capturing of fluorescence images. Contrast and brightness of images were adapted with CellF. Figure compilation was done with Powerpoint (Microsoft), and high quality tif-formats were produced by Photoshop (Adobe).

### Column fractionation of milk and milk cell samples

All protocols steps were performed with cooled solutions and cooled equipment kept at 4 °C. Milk samples were diluted with an equal volume of cooled phosphate buffer saline (PBS) (pH 7.2), and centrifuged for 10 minutes at 500 g to obtain milk cells, skimmed milk and fat fractions. The fat and skimmed milk fractions were then transferred into new tubes. The skimmed milk fraction was centrifuged for a second time to remove remaining cell and fat components. The skimmed milk fraction was then loaded on a self-poured Sephadex G-75 column (Sigma-Adrich, G75120). The column was washed with 30 ml PBS and 1 ml fractions of flow through were collected, and labelled F1 to F30. For isolation of the fluorophores from the sedimented cells, the cell pellet was washed in 5 ml PBS and centrifuged again. The milk cells were then dissolved in PBS and extracted by sonication (Bandelin Sonopuls equipped with a sonication tip). Remaining particles were removed by centrifugation (5 min, 4000 g). The supernatant was transferred on a pre-wetted tip500 anion exchange column (Qiagen). The column was washed with 30 ml PBS and 1 ml fractions of flow through were collected.

### Signal peptide analysis and glycosylation site prediction

The polypeptide sequences of Venus, mCherry and porcine caseins were analysed by the Signal4.1 software (http://www.cbs.dtu.dk/services/SignalP). SignalP4.1 predicts the presence and location of signal peptide cleavage sites in amino acid sequences for different organisms. The method incorporates a prediction of cleavage sites and a signal peptide/non-signal peptide prediction based on a combination of several artificial neural networks.

For the prediction of N- and O-glycosylation sites the NetNGlyc 1.0 Server (www.cbs.dtu.dk/services/NetNGlyc) and the NetOGlyc 4.0 Server (www.cbs.dtu.dk/services/NetNGlyc) were used.

### Deglycosylation assay

To determine potential glycosylations, skimmed milk fractions of the milk from Venus and mCherry sows were treated with O-glycosidase/neuraminidase, PNGase F and a combination of O-glycosidase/neuraminidase and PNGase F (New England Biolabs) according to the recommendations of the manufacturer. The treated samples were then used for SDS-PAGE, blotted and incubated with specific anti-GFP and mCherry antibodies. As control, a glycosylated fetuin sample (NEB) was deglycosylated and detected by Coomassie staining after SDS-PAGE.

## Additional Information

**How to cite this article**: Mukherjee, A. *et al.* Expression of Active Fluorophore Proteins in the Milk of Transgenic Pigs Bypassing the Secretory Pathway. *Sci. Rep.* **6**, 24464; doi: 10.1038/srep24464 (2016).

## Supplementary Material

Supplementary Information
