# Supplementary material for: Expression of Active Fluorophore Proteins in the Milk of Transgenic Pigs Bypassing the Secretory Pathway
Source: Sci Rep. 2016 Apr 18;6:24464. doi: 10.1038/srep24464 (PMC4834472; doi:10.1038/srep24464)
Supplement: Supplementary Information [file srep24464-s1.pdf]

Supplementary Information

Expression of Active Fluorophore Proteins in the Milk of Transgenic Pigs Bypassing the Secretory Pathway

Ayan Mukherjee[1], Wiebke Garrels[2], Thirumala R. Talluri[1, #], Daniela Tiedemann[1], Zsuzsanna Bösze[3], Zoltan Ivics[4], Wilfried A. Kues[1,*]

[1]Friedrich-Loeffler-Institut, Institut für Nutztiergenetik, Mariensee, Germany;
[2]Medical School Hannover, Institute of Laboratory Animal Sciences, Hannover, Germany;
[3]Agricultural Biotechnology Center, Gödöllö, Hungary;
[4]Paul-Ehrlich-Institute, Langen, Germany.

Short title: Fluorophores from transgenic milk

Key words: Bioreactor, Recombinant protein, Secretory pathway, Exfoliated cell, Signal peptide, Sleeping Beauty transposition.

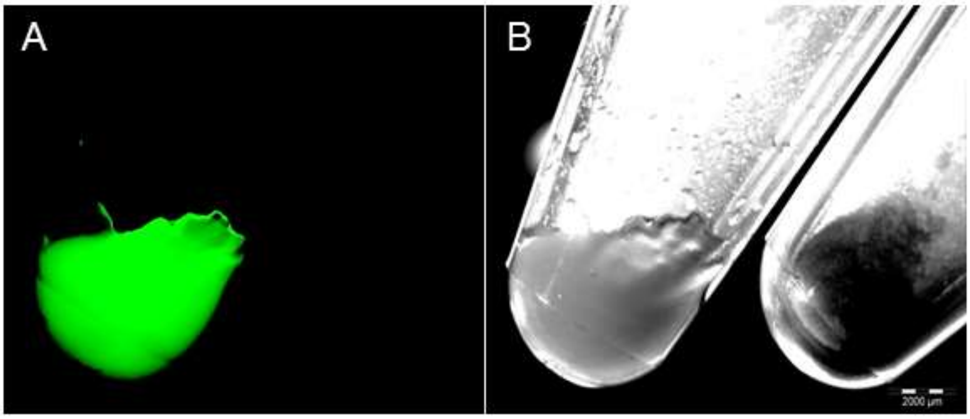

Supplementary Fig. S1. Purified Venus protein

A) Dried fraction F10 (left) and dried non-transgenic milk powder (right) shown under specific excitation of Venus. The tube contains 200 μg of Venus protein, lyophilised from 500 μl of fraction F10 (Fig.2g).

B) Corresponding white light image. The samples are on the bottom of standard 1.5 ml centrifuge tubes.

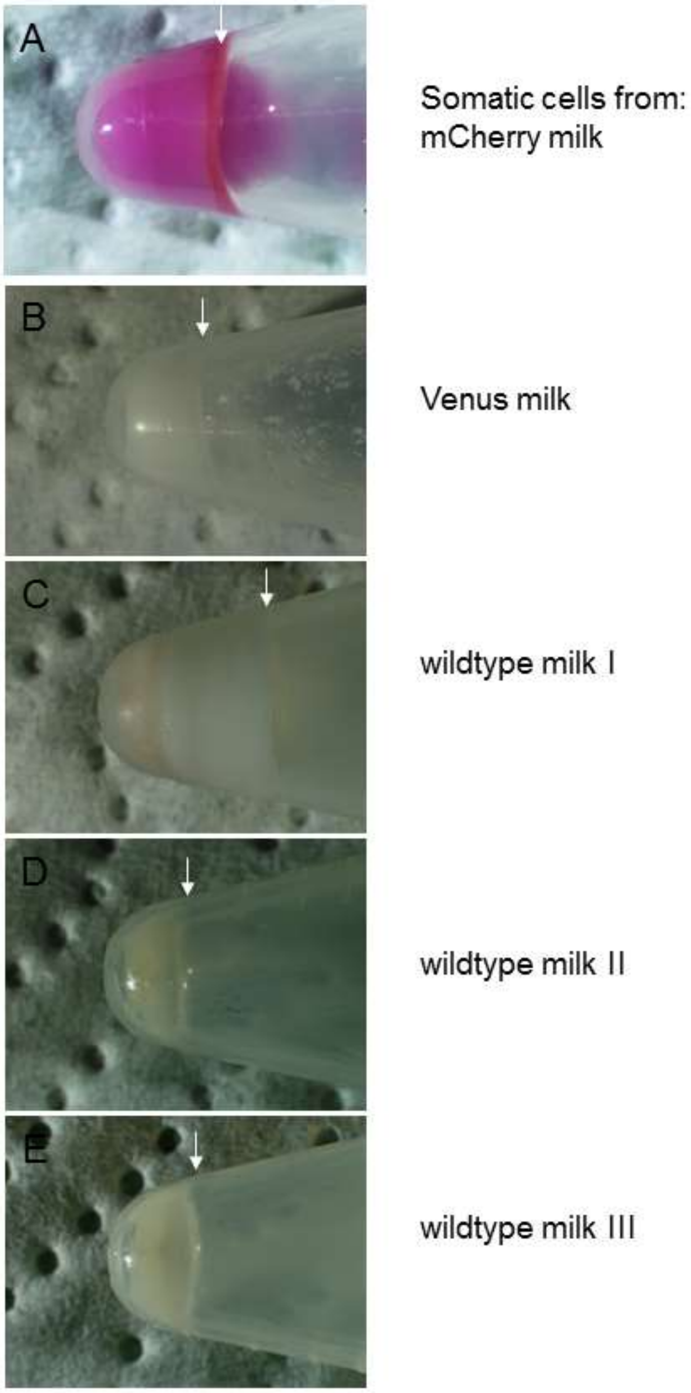

Supplementary Fig. S2 Amount of exfoliated cells in sow milk

Identical volumes (15 ml) of sow milk at midlactation (d10-d15) from A) a mCherry transgenic sow, B) a Venus transgenic sow, and C-E) three wildtype sows were centrifuged. The amounts of the cell pellets are indicated by arrows.

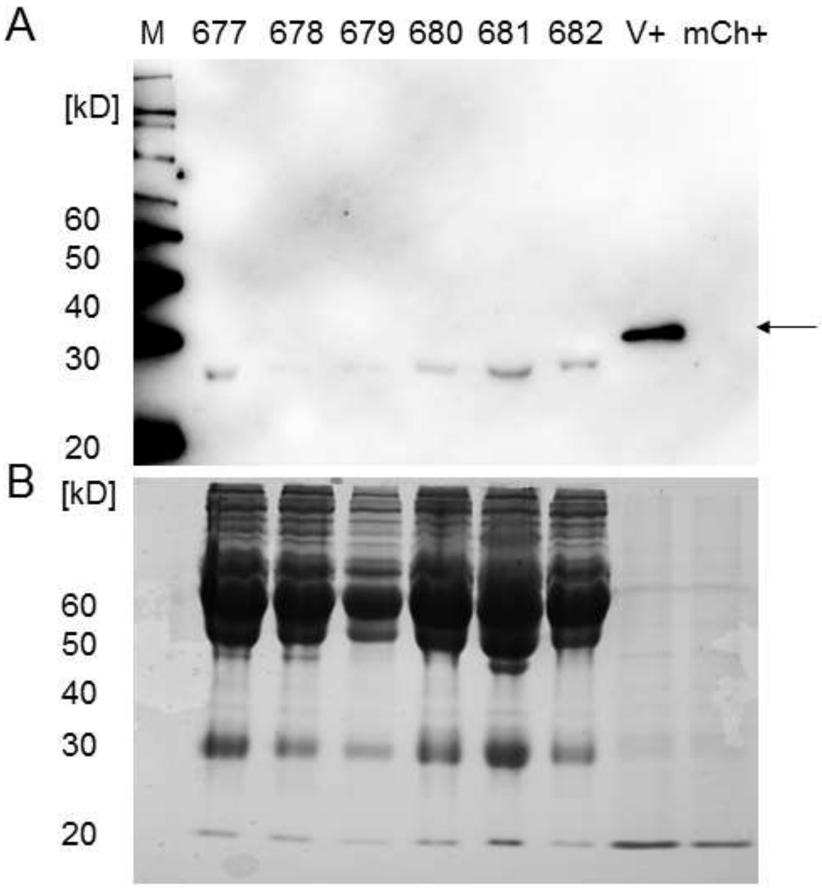

Supplementary Fig. S3. Absence of Venus protein in blood plasma

A) Plasma fractions of 5 Venus transposon animals (#677 - #681), a non-transgenic littermate (#682), and milk samples from a Venus (V+) and mCherry (mCh+) transposon sows were probed with an anti-Venus antibody.

B) Corresponding Coomassie stained gel.
